# An Evidence-Based Procedure for Self-Management of Medication in Hospital: Development and Validation of the SelfMED Procedure

**DOI:** 10.3390/pharmacy6030077

**Published:** 2018-07-26

**Authors:** Toke Vanwesemael, Tinne Dilles, Bart Van Rompaey, Koen Boussery

**Affiliations:** 1 Department of Healthcare, Thomas More University College, 2500 Lier, Belgium; tinne.dilles@uantwerpen.be; 2 Department of Nursing Science and Midwifery, Centre For Research and Innovation in Care (CRIC), Nurse and Pharmaceutical Care (NuPhac), Faculty of Medicine and Health Sciences, University of Antwerp, 2610 Wilrijk, Belgium; bart.vanrompaey@uantwerpen.be; 3 Pharmaceutical Care Unit, Faculty of Pharmaceutical Sciences, Ghent University, 9000 Ghent, Belgium; koen.boussery@ugent.be

**Keywords:** hospital medicine, medication management, medication self-management, inpatients

## Abstract

Aim: To develop and validate a procedure for self-management of medication by patients whilst in hospital. Background: Self-management of medication allows patients to self-manage their medication in a controlled and supportive hospital environment. This practice is encouraged worldwide, yet an evidence-based procedure to evaluate the ability of patients to self-manage and to monitor and support self-management are absent. Methods: The evidence-based procedure for self-management of medication (SelfMED) was developed based on previous conducted qualitative research, literature review, and the current regulation. It was validated by healthcare providers and a multidisciplinary expert meeting. Questions within the procedure that could be biased were tested for inter-rater reliability. Results: First, the SelfMED procedure was developed. It consists of a stepped assessment of patient’s competencies for self-management performed by healthcare providers and the patient. When self-management is allowed, the SelfMED monitoring tool monitors the patient’s intake of self-managed medication. Secondly, the procedure was revised for clarity, appropriateness, and face validity by five healthcare providers and a multidisciplinary expert meeting, resulting in the final version. Thirdly, three questions from the final version were tested for interrater reliability. Cohen’s Kappa showed moderate to strong levels of agreement. Conclusions: The developed SelfMED procedure provides an evidence based approach of facilitating self-management of medication. The content of the procedure was found valid to evaluate the patient’s ability to self-manage and to monitor them while self-managing.

## 1. Introduction

Patients self-administering their medication while hospitalized has been mentioned in literature as far back as 1959 [1]. Previous research established self-administration of medication has been implemented in acute hospitals in the United Kingdom. Nevertheless, the levels of implementation remained variable [2,3]. The Society of Hospital Pharmacists of Australia, described self-management as a strategy to evaluate the medication management of hospitalized patients in order to prevent medication related problems after discharge. Facilitating self-administration in a supervised setting and combined with support could result in confident and competent patients. The Society describes self-administration in hospitals as an important contribution to a return to independent living at home after discharge [4]. Belgian research on the actual prevalence showed 22% of hospitalized patients did self-administer medication. Concerning the opinion of nurses on the ability of these hospitalized patients to prepare and administer medication by themselves, just 40.9% of the patients were deemed able to do so. The decision of allowing self-administration was mostly a shared process between patients and healthcare providers. In 28.3% the patient, nurse, and treating physician were involved and in 26.3% the nurse and the patient [5]. Overall, it is possible to conclude self-management is not an unusual practice and it has been studied and implemented worldwide [4,6,7,8].

Although, self-administration of medication is implemented in practice, a rigorous study on 56 Flemish nonacute hospital wards showed some clear shortcomings. Only 17.9% of the included wards had a procedure for self-administration of medication and 7.1% of the wards had a screening tool to assess patients on their self-management ability before allowing them to self-administer [5]. A qualitative study on the perspectives of healthcare providers and patients concerning self-administration of medication during hospitalization acknowledged these findings. The interviewed respondents indicated it is not clear how to identify whether a patient is able to self-administer medication or not. Also, healthcare providers and patients worried about how to monitor self-administered medication correctly, as there was no guideline to support this act. Respondents in this study were not up to date on the current legislation or regulation concerning self-administration of medication and were very insecure on how to handle this in practice [9].

Self-administration of medication, as described in the literature, focuses on the actual administration of medication. However, allowing self-administration of medication by patients whilst in hospital requires adjustments in general medication management (i.e., preference for the use of unit dose medication and providing patient education on self-administered medication). Therefore, the term self-management of medication is preferred. This term includes both medication administration and aspects of medication management such as monitoring self-administration, providing education on self-administered medication by healthcare providers, and the support of every stakeholder in the process in order to succeed in self-managing medication [5,6,9]. Two systematic reviews on self-management of medication confirmed the presence of very diversely structured self-management programs (SMP’s), with different contents. Although, several SMP’s have been designed in studies, very little have been thoroughly validated and tested [6,7].

As previous research showed, a self-management of medication procedure should include an assessment in order to define whether a patient is capable of self-managing medication. If a patient is deemed capable, a monitoring tool should support self-management to be safe and all aspects on the current regulation should be clear in order to adhere to [5,9].

A literature search on assessments in order to define whether a patient is capable of self-managing medication identified two articles on the validation of a self-administration of medication (SAM) screening tool in order to define the patient’s competencies. The SAM screening tool consisted of two parts. The first part had to be answered by the patient and was filled in by an administrator. The patient’s desire to self-manage was evaluated with the use of a visual analog scale. This scale questioned the patient (1) whether he/she deems him/herself competent for managing regular medication independently while in hospital and (2) how much he/she would like to manage these regular medications while in hospital. Also, demographic data on the patient’s discharge destination and responsibility for medication management following discharge was collected. The second part had to be filled in by the nurses if the patient was willing to self-manage. It consisted of questions on the patient’s capability to self-medicate (11 questions), knowledge of medications and behavior (7 questions), and experience with self-medicating (6 questions). In the end, the nurse needed to make a global assessment with the use of a visual analog scale, resulting in a maximum score of 96 points. The cut-off was installed at 60, below this cut-off patients were not able to self-manage medication [8,10]. Specific literature concerning the validation of a monitoring tool to follow up self-administration are still lacking, although the systematic review written by Wright et al. indicated nurses monitored the intake of self-administered medication. Yet, monitoring in these studies was part of collecting the outcome (adherence), not a way of observing and monitoring self-management [6,7].

Results from previously conducted research provided some evidence on the content of a procedure for self-management of medication in hospitals. Nevertheless, they revealed some important issues not included in the previous tools. First, there is a need for a multidisciplinary approach; hospital pharmacists, physicians, nurses, and patients have their own responsibilities and should therefore be included in the process of evaluating competencies for self-management [11]. Secondly, this multidisciplinary approach should be included in a much-needed assessment which clarifies and takes into account the current legal framework on self-management in hospitals. Thirdly, an instrument for monitoring self-management should be provided within the procedure of self-management. Therefore, this study aimed to develop and validate a procedure for self-management of medication whilst in hospital, also named the SelfMED procedure.

## 2. Materials and Methods

During the first stage of this study a procedure for self-management of medication was developed. During the second stage, it was validated with the involvement of several healthcare providers and a multidisciplinary expert meeting. Questions in the assessment that could be biased by the opinion of nurses were tested for inter-rater reliability. The study was conducted in accordance with the Declaration of Helsinki, approval of the ethics committee of the general hospital Klina was provided on 19 November 2015, reference number 031/200/015.

### 2.1. Stage 1: Development of Procedure

During the first stage of developing the SelfMED procedure several actions were undertaken. As shown in Figure S1, a previously conducted qualitative study on the perspectives of patients, nurses, physicians, and hospital pharmacists concerning the strengths, weaknesses, opportunities, and threats of self-management of medication during hospitalization was used. Study results indicated both healthcare providers and patients stated important prerequisites in order to facilitate self-management of medication in hospital. Analysis of these results allowed the authors to allocate these prerequisites to three major topics; prerequisites related to the patient, the medication, and the organization. Those related to the patient were; i.e., patients had to be self-managing medication at home before hospitalization, they had to be willing to self-manage medication, and had to be able—mentally and physically—to self-manage medication. Those related to the medication were; i.e., self-managed medication should not consist of to many different types of medication. Those related to the organization were; i.e., provide a clear legal context with defined responsibilities in case of self-management, and a system to monitor medication self-management [9]. Secondly, literature on medication adherence and validated tools to assess patient’s competencies to self-manage were supplementary examined and compared to the findings of the qualitative research. Also, the Morisky Medication Adherence Scale was consulted [8,9,10,12]. Thirdly, it was important to adhere current regulation concerning medication self-management in Belgian hospitals. Therefore, the Care inspection of the Flemish division of Wellbeing, Public Health, and Family was consulted. Self-management of medication is allowed in hospitals if the self-managed medication is registered in the patient’s personal medical file. During this process, healthcare providers maintain their duty of care and their duty of surveillance, while the treating physician takes responsibility for allowing and evaluating self-management of medication (Care inspection of the Flemish division of Wellbeing, Public Health, and Family, personal communication, October 2015). Because of the current regulation on self-management, the developed SelfMED procedure clearly stated self-managed medication had to be noted down in the patient’s medical file. Also, the SelfMED monitoring tool—a part of the SelfMED procedure—facilitated the duty of surveillance and care, and the overall evaluation of self-management. In the end, the described role of treating physician in the procedure indicated he/she has the final decision on allowing or declining self-management and the type of self-managed medication.

### 2.2. Stage 2: Validation

The first draft of the SelfMED procedure was validated in several phases (see Figure S1), by three pharmacists, a physician, and a nurse manager who were employed in both university and regional hospitals. They were selected based on their knowledge of hospital medication management, tool development, and their role in direct patient care.

The first draft was evaluated on clarity, appropriateness, and face validity. Face validity measured the degree to which the included questions measured what they were supposed to [13,14]. Also, the clarity and wording were evaluated during this process. In addition, the healthcare providers assessed the importance attached to the content of the questions and the appropriateness of the response scale used. The format and the overall presentation of the SelfMED procedure was evaluated. After the evaluation, adjustments were made based on the feedback; fewer answer categories were provided and questions were reformulated in order to avoid jargon in the patient’s self-assessment. The monitoring tool for self-managed medication was adjusted in order to make it easier to use and a clear description was provided to support completing the form correctly.

The second draft of the SelfMED procedure was discussed in a multidisciplinary expert meeting. The expert meeting consisted of nurses (n = 3), a nurse manager (n = 1), physicians (n = 2), and a hospital pharmacist (n = 1) from a regional hospital. They were selected based on their knowledge on hospital medication management in daily practice, as stakeholders who would use the procedure in daily practice. Again, a revision for clarity, appropriateness, and the format and overall presentation was conducted. Some minor alterations were made after this expert meeting; the lay-out was simplified and adjusted in order to facilitate the use of a paper-based SelfMED procedure, the actual number of days a patient needed to be hospitalized in order to assess his/her ability to self-manage was deleted, the order of the first two statements of the nurse assessment was changed, the provided description on how to use the monitoring tool was rewritten more concisely.

A part of the SelfMED procedure consisted of an assessment that questioned the opinion of a nurse on the capabilities of hospitalized patients to self-manage their medication. Three statements concerned the mental and physical state of the patient, and one the capability to deal with changes in the medication regimen. As these questions may be biased by the nurse assessing the patient, two nurses independently assessed patients.

### 2.3. Study Methodology

The study on evaluating bias took place in a regional hospital, on a cardiology ward specialized in heart failure, cardio revalidation, and postinterventional care. Two nurses agreed on assessing the patients, both nurses were working on the participating ward. All consecutive patients on the ward were eligible to participate if over the age of 18 years old. All subjects received oral and written information about the study and who—with the use of the nurse’s assessment—assessed their competence for self-management of medication in hospital. Also, the nurse’s assessment evaluated whether patients had to be taking medication in hospital, if not they could not participate the study. All patients gave their informed consent for inclusion before they participated in the study. Data collection was performed from February until March 2016. During the completion of the assessment there was no contact between the assessing nurses. Both conducted their assessment one after the other, in order to rule out possible changes in the health status of the patients. Afterwards the inter-rater reliability was calculated by the Cohen’s Kappa, this resulted in the percentage agreement between both nurses [15,16,17,18]. The Statistical Package for Social Sciences (SPSS) version 24.0 (SPSS Inc., Chicago, IL, USA) was used to analyze the data. Discontinuous data were described using frequency distributions. Continuous data were described using a mean value and standard deviation if normally distributed or using a median and range if non-normally distributed. A *p* value of 0.05 was considered as statistically significant.

### 2.4. Translation

For publication purposes a forward-translation from the Dutch version of the SelfMED procedure to English was performed. Two translators independently performed their translations, one translator was a native English speaker the other was perfect bilingual. None of them were previously involved in this study, yet they did have knowledge concerning medication management, healthcare, and hospital care. After translating, both translators discussed their translations and resolved some differences. Then, the English version of the SelfMED procedure was back-translated by an independent translator with an academic background and a level five (C1) in English concerning the Common European Framework of Reference. This translator was bilingual and has not been involved in this, nor previous studies concerning this topic. Back-translation revealed some minor unclear wordings; these were adapted in the English version [16].

## 3. Results

### 3.1. Stage 1: Development

The SelfMED procedure in this study was developed through several stages (see Figure S1). As a result of this process, the SelfMED procedure consists of several phases, each described in following sections (see Figure S2). The complete SelfMED procedure can be consulted in Figure S3.

#### 3.1.1. Nurse Assessment

A ten-statement assessment allows the nurse to assess the eligibility of the patients for self-management of medication. Filling out this assessment can be done based on the information which is obtained during the intake and information available in the patient’s medical file. Nurses are able to define an answer to the statement in the first column (agree/not agree/not known) and assess whether the statements are a barrier for self-management in the second column (barrier for self-management/possible barrier for self-management/no barrier for self-management). At the end of the assessment the nurse indicates whether the patient is eligible for self-management, if so the patient needs to further fill out the patient self-assessment.

#### 3.1.2. Patient Self-Assessment

If the nurse deems the patient capable of self-managing medications, the next phase of the procedure consists of a patient questionnaire. The patient should complete this self-assessment on their own. The questions in the assessment consist of: the current medication management at home (one item), the patient’s willingness to self-manage medication in hospital (one item), a possible need for aide while self-managing in hospital (one item), and the patient’s therapy adherence out of hospital (seven items). If a patient indicates he/she is not self-managing medication at home or is not willing to self-manage medication, he/she does not have to complete the questionnaire.

The information from both the nurse’s assessment and the self-assessment enables nurses to formulate advice for the treating physician to allow or decline self-management of medication. If patients are willing to self-manage medication, nurses can further interpret the results of the resulting questions concerning the patient’s medication management at home and their medication adherence. Because of the active involvement of the patient during the assessment, it is possible to also test the patient’s fine motor skills. These skills are of great importance in, for example, opening medication blisters or checking off the medication list of self-managed medication.

If the nurse formulates negative advice, there is a possibility to advise a reassessment at another moment in time. Improvement in health status can result in improvements in self-management competence.

#### 3.1.3. Physician Assessment

In the next phase, the patient’s physician can make a final decision on allowing the patient to self-manage medication. The advice formulated by the nurse, the nurse’s assessment, and the patient’s self-assessment are available to make an informed decision. If the treating physician decides it is not allowed to self-manage medication, there was a possibility to re-assess the patient at another moment in time. If a patient is judged to be eligible and self-management is allowed, the actual self-managed medicines need to be specified by the treating physician and registered in the patient’s personal medical file. A clinical pharmacist can be involved to provide supplementary comments if they are involved in the medication process.

#### 3.1.4. Practical Issues for Starting Self-Management of Medication

If a patient is capable of self-managing medication, several practical arrangements have to be made: provide the patient with a medication list on his medication, provide correct medication to the patient room, and document the self-managed medication in the patient’s medical file. Also, patients need to be educated on the monitoring system.

#### 3.1.5. Monitoring Self-Management of Medication

Self-managing patients are instructed on the use of the monitoring tool. They are aware of the type of medication, the time when to take medication, and the dosage. If the self-managed medication has been taken, patients are asked to check off these medicines on the hour of intake on their medication list. On a daily basis—during medication administration tours—the list with self-managed medication has to be checked for mistakes by the nurse. If problems concerning medication self-management occur, there is doubt about medication intake, or for research purposes, a pill count can be provided optionally in order to detect medication errors.

The monitoring tool consists of a first column to define the date and the second shows the initials of the monitoring nurses that day. The third and fourth column questions whether the patient is still capable to self-manage medication, if not the reason should be formulated (i.e., sudden illness, mental decline). If the status of the patient has not changed and he/she was self-managing medication, the medication list is then evaluated and a pill count is optionally performed. The results should be noted in the monitoring file on the patient’s room. If the patient succeeds in every aspect of self-management, self-management can be continued. If patients fail in self-managing their medication, the nurse can intervene by providing patient education to prevent this error from occurring again. If the errors in the self-management are found to be problematic, it is possible to stop self-management. In case a patient is not capable of self-managing medication, self-management stops. A possibility to reassess over a period of time is provided.

### 3.2. Stage 2: Validation

Question four, five, and seven in the nurse’s assessment may be biased by the rater (see section Nurse assessment Figure S3). Therefore, the kappa statistic was used to test inter-rater reliability [14]. A total of 158 hospitalized patients were assessed by two raters. Table 1 gave an insight in the demographics of the assessed patient population. Only, if the assessing nurse deemed patients capable, they completed the self-assessment. Therefore, only data from these patients on the level of education, chronic medication intake at home, and self-management of medication during previous admissions were collected and showed in Table 1. As shown in Table 2 all three questions had moderate to strong levels of agreement between both raters (n = 158, question 4 κ 0.892, question 5 κ 0.843, question 7 κ 0.784; *p* < 0.001).

## 4. Discussion

In this study the SelfMED procedure was developed. This procedure guides and supports patients and healthcare providers during the decision-making process concerning allowing or declining self-management and during actual self-management of medication. During the development, the opinion of, not only nurses and patients, but also hospital pharmacists and physicians and the current regulation on self-management in hospitals were taken into account. Therefore, all these important stakeholders were included in the procedure.

The SelfMED procedure consists of a stepped assessment performed by the nurse (1), the patient (2), and the hospital pharmacist and treating physician (3). This assessment enables the treating physician to provide a well-informed decision on allowing or declining a patient to self-manage medication in hospital. If a patient is allowed, the SelfMED monitoring tool will monitor the intake of self-managed medication and detect possible medication errors or other medication related problems. If problems occur, the SelfMED procedure encourages interventions such as patient education to provide them from reoccurring. The procedure distinguishes itself from previously developed tools, as it includes all important stakeholders and adheres to current regulation. Furthermore, it not only assesses patient’s competencies but also guides and supports monitoring self-management and encourages healthcare providers to improve the patient’s self-management skills if necessary [6,7,10].

The role of hospital pharmacists was briefly described in the assessment phase. Nevertheless, their involvement is foremost important. As described in literature, the active involvement of hospital pharmacists on clinical wards resulted in several benefits such as improved care and reduced harm [19]. Specifically, for self-management of medication in hospital, hospital pharmacists can provide counseling sessions for patients when problems arise concerning adherence that occurs during self-management, and they can support nurses in educating patients on medication. Also, pharmacists can clarify discharge prescriptions, as literature indicated this as a problem for patients [20]. Research on hospital pharmacists providing patient counseling before hospital discharge and telephone follow up after discharge indicated a significant association with less adverse drug events [21]. These findings strengthen the hospital pharmacists’ important role.

### 4.1. The SelfMED Procedure in Daily Practice

Previous literature on self-management of medication indicated possible advantages such as; increased patient satisfaction, increased patient safety, an improvement of adherence to pharmacotherapy, and self-care competence [5,6,9,22]. Research on the willingness of patients highlighted they were generally very willing to self-manage medication in hospital. Nevertheless, if patients do not want to self-manage medication this should be respected [22].

Notwithstanding the possible advantages of self-management of medication, actually implementing the SelfMED procedure requires some changes in the current medication management process. Compared to nurses preparing and administering medication, self-management implicates patients have to be assessed first to determine their competence for self-management. Additionally, self-managed medication is ideally supplied as unit dose and logistics on how to transport medication to the patient room and store it should be apparent. Previous research already provided a flowchart on these advised adaptations [9]. Secondly, problems on medication shortages might influence the delivery of self-managed medication, as the medicines might not be in stock [23].

Also, adaptations on ward-level are advisable. As stated in the procedure, the storage of self-managed medication in closed lockers or the patient’s personal locker is advised. It is possible not all patient rooms have access to a personal locker. Nevertheless, nightstands mostly tend to have a build in locker. Also, when self-managing patients check off their medication list, as stated in the SelfMED procedure, nurses are not possible to observe this other than checking the actual medication list on the patient’s room. It would be an added value if this medication list could be provided on a tablet or linked to an electronic patient data management system. Additionally, this can also provide nurses with an overview on self-managed and nurse administered medication.

### 4.2. Implications for Research

The SelfMED procedure developed and validated in this study provides a first evidence-based guide for self-management of medication. In order to refine and provide further improvements, following implications for research were discussed.

The current SelfMED assessment comprised a stepped approach with a limited amount of questions for nurses, patients, and physicians. It is expected that due to this approach the time investment for completing the assessment remains limited for each stakeholder. Supplementary research on the effect of self-management on time management of involved stakeholders is advised.

Because of the possible bias in three questions from the nurse assessment, inter-rater reliability was calculated. Yet, in future research the inter-rater reliability of the complete SelfMED assessment should be determined, since the opinion of physicians can be a subject to bias and bias in other questions from the nurse assessment could be present.

When patients are found to be capable to self-manage, the nurse formulates a positive advice for the treating physician. It is expected nurses are adequately educated and have sufficient clinical judgment to assess a patient with the use of a checklist already providing all important topics. By providing these topics, reproducibility can be increased. The final decision on self-management can be taken by the physician. As this healthcare provider remains responsible for medication during hospital admission, the physician will be able to define which medicines can be self-managed. Previous qualitative research indicated physicians are more likely to prohibit patients to self-manage high-risk medication. Nevertheless, this viewpoint can be discussed and should be investigated further, for example transplant patients self-manage their medication in order to be able to manage them correctly after discharge. Allowing patients to already self-manage high-risk medication in a controlled environment might ultimately result in a benefit for the patient [9]. Literature on the treatment of diabetes in hospital even encouraged patients to self-manage their insulin when hospitalized on the condition that this is also monitored by healthcare providers [24,25].

The current SelfMED monitoring tool provides an evaluation of medication self-management. If medication errors occur, the flowchart suggests healthcare providers to educate patients in order to prohibit the error from reoccurring. As this is a crucial step towards improving the self-management skills and possible medication knowledge further research should focus on expanding this aspect of the SelfMED procedure [26].

Previous literature on the effect of self-management of medication indicated several positive results. Nevertheless, these results should be taken into account cautiously due to methodological flaws and low-quality research [6,7]. Future research should therefore focus on the effect of self-management of medication on patient outcomes (patients satisfaction, medication errors during hospitalization, influence on therapy adherence after discharge, and patient self-management skills after discharge), the influence on quality of care, medication management processes, and influence on the healthcare costs.

The current SelfMED procedure was developed focusing on the average hospitalized patient on a cardiology ward. Therefore, it is important when using this procedure in a specific population, in example geriatric or psychiatric patients, adaptations in the assessment criteria might be needed in order to meet the aim of correctly assessing patients and supporting self-management of medication.

## 5. Conclusions

The SelfMED procedure developed and validated in this study has the potential to guide and support self-management of medication in hospital. Because of the inclusion of all important stakeholders within the medication management process, the evidence-based approach and the fit with current regulation the procedure distinguishes itself from previously described tools. Further refinements and validation in daily practice are advised, also a tool for providing tailor made interventions for medication related problems during self-management should be developed and validated.

## Figures and Tables

**Table 1:** Demographics.

Patient Characteristics	n	%
Age (yr, mean [range])	158	72.8 [23–95]
Sex	158	
Male		47.5
Female		52.5
Level of education	73	
None		12.3
Primary school		17.8
Secondary school		52.1
Bachelor		15.1
Master		2.7
Chronic medication intake at home (mean [range])	71	4.7 [0–15]
Self-management of medication during previous admissions	74	
Yes		74.3
No		25.7

**Table 2:** The inter-rater reliability of three question from the nurse’s assessment.

		Opinion Nurse 2 (n)			
		Not agree	Agree	Unknown	Kappa *p* value
	Opinion nurse 2 (n)				
The patient is physically able to self-manage his medication (n = 159)	Not agree	33	0	0	0.892 < 0.001
	Agree	6	120	0	
	Unknown	0	0	0	
The patient is mentally able to self-manage his medication (n = 159)	Not agree	46	1	0	0.843 < 0.001
	Agree	10	102	0	
	Unknown	0	0	0	
The expectation is that the patient can handle possible treatment changes (n = 159)	Not agree	47	3	0	0.784 < 0.001
	Agree	10	96	0	
	Unknown	3	0	0	

## Supplementary Materials

The following are available online at http://www.mdpi.com/2226-4787/6/3/77/s1, Figure S1: Development and validation of the SelfMED procedure, Figure S2: The SelfMED flowchart, Figure S3: The SelfMED procedure.
