# Supplementary material for: An Evidence-Based Procedure for Self-Management of Medication in Hospital: Development and Validation of the SelfMED Procedure
Source: Pharmacy (Basel). 2018 Jul 26;6(3):77. doi: 10.3390/pharmacy6030077 (PMC6164845; doi:10.3390/pharmacy6030077)
Supplement: Supplementary file 1 [file pharmacy-06-00077-s001.zip › Supplementary Files/Figure S3.pdf]

1 Nurse assessment

This assessment is to be completed by the nurse, who, based on a series of set criteria, will evaluate if the patient can manage their own medication during hospitalisation.
For each question, there are three possible options:
If the statement is

- not correct, then check the 'not agreed' box
- is correct, then check the 'agreed' box
- is not known, then check the 'not known' box

In addition to the statement there will also be an evaluation if there is a possible contradiction for medication self-management.

	**Statement**			**Consequences for self-management**		
	Not known	Not agree	Agree	Contra-diction	Possible Contra-diction	No contra-diction
1 The patient can prepare his/her own medication at home.						
2 The patient administers his/her own medication at home.						
3 After release from hospital the patient is capable to prepare and administer his/her own medication.						
4 The patient is physically able to administer his/her own medication (for example health conditions, physical restrictions, ability to open the medication, …).						
5 The patient is mentally capable of controlling his/her own medication (mental confusion, understands the purpose of hospitalisation, …).						
6 I have no knowledge of substance abuse by the patient (history check, …)						
7 The expectation is that the patient can handle possible treatment changes (reduction in medication, adjustments to new medication, ….).						
8 The patient will not be exposed to any other medical interventions (i.e. surgery) where a new medication scheme will need to be implemented by nurses.						
9 The expectation is that the hospitalisation will be long enough to allow the patient to commence his/her own medication therapy.						
10 The patient speaks sufficient Dutch to understand the treatment (verbal, written).						
11 ……						

Result nurse assessment:

Perform self-assessment:	O No→ 0 No re-evaluation<br>O Yes	0 Re-evaluate on ……. / …… / …… (date)

Figure S3: The SelfMED procedure.

2 Self-assessment

The following questions need to be answered by the patient which will be included in the assessment

1 How do you administer your medication at home?

- o I can prepare and administer my medication
- o The medication is prepared for me (i.e. using pre-filled medication dosage) but I am able to administer it myself
- o The medication is prepared for me (i.e. using pre-filled medication dosage) and I need help with administering the medication (i.e. the medication is administered by family, nurses or caregivers) → if this is applicable you don't have to complete the questionnaire

2 Would you be prepared, with support from caregivers, to control your own medication during your hospital stay?

- o Yes, I would
- o No, I would not → if this is applicable you don't have to complete the questionnaire

3 Do you believe, with support and control from caregivers, you can manage your own medication during your hospital stay?

- o Yes
- o Yes, with support from/or with help of: ……………………………………………………………….
- o No

4 Do you sometimes forget to take your medication? Check the correct answer

o Never o Sometimes o Frequently o Usually

5 People sometimes miss taking their medications for reasons other than forgetting. Thinking over the past two weeks, were there any days when you did not take your medicine?

o No, I took my medication correctly o Yes, I missed taking my medication once o Yes, I frequently missed taking my medication o Yes, I always missed taking my medication

6 Have you ever cut back or stopped taking your medicine without telling your doctor because you felt worse when you took it?

o Never o Sometimes o Frequently o Usually

7 When you travel or leave home, do you sometimes forget to bring along your medicine?

o Never o Sometimes o Frequently o Usually

8 When you feel like your symptoms are under control, do you sometimes stop taking your medicine?

o Never o Sometimes o Frequently o Usually

9 Taking medicine every day is a real inconvenience for some people. Do you ever feel hassled about sticking to your treatment plan?

o Never o Sometimes o Frequently o Usually

10 How often do you have difficulty remembering to take all your medicine?

o Never o Sometimes o Frequently o Usually

Figure S3: The SelfMED procedure.

3 Recommendation for the patient's self-medication

Based on the assessment of the nurse and the patient's self-assessment the following is recommended. For a negative assessment, there is then a recommendation for the timescales for any follow-up assessment (for example 2 days after an operation).

Recommendation:

- The patient is not able to control self-medication.
- The patient is able to control self-medication. Recommended support……………………………………………………………………….

Re-evaluation of recommendation…

- No further re-evaluation during current hospitalization.
- Re-evaluate on: ...... /....... /......... (date)

Remarks:

………
………

Figure S3: The SelfMED procedure.

4 Physicians assessment

Based on the recommendation from the nurse, and if necessary the patient's self-assessment, the doctor will make the following guidance on the patient undergoing self-medication. These recommendations can be complimented by comments from the clinical pharmacist, where they have been involved in the medication process.

Advice:

- o The patient is not able to control self-medication.
  Reason: ………..
- o The patient is able to control all his/her self-medication. Recommended support: …………………………………………………….
- o The patient is able to control a part of his/her self-medication. Recommended support: ………………………………………………

Re-evaluation of advice…

- o No further re-evaluation during current hospitalization.
  Reason: ………..
- o Re-evaluate on: ...... /....... /......... (date)

Where the decision is made to proceed with self-medication the following table needs to be completed listing the name of the medication and the details of the medication program. Any future change, or adjustment, in medication must be recorded.

Name medication to be administered by the patient	Dose	Method	Frequency

Figure S3: The SelfMED procedure.

5 Practical issues for starting self-management of medication

It is important to note down overall attention points and agreements around self-management of medication. Below mentioned items will be a guideline for the execution of the self-management.

- Create a checklist in consultation with the ward on all topics that need to be provided when patients start to self-manage their medication: note down in the patient's medical file which medication will be self-managed, provide a medication list for the self-managed medication, order self-managed medication from the hospital pharmacy, provide education on the self-managed medication and the use of the medication list.

- Medication can be self-managed (preferably in closed lockers, personal locker, nightstand) and self-administered by the patient. The allowed self-managed medication needs to be discussed with the relevant department, treating physician and hospital pharmacist.

- On a daily base the patient needs to register on the medication list per medicine the dose and the timing of the medication intake. It's allowed that the patient uses additional tools like a clock or an alarm to remind him of the medication intake.

- It is important that the patient immediately gets a new medication list when adjustment/updates are needed.

- The medication lists who are not correct or outdated, will be stored in a specific place as agreed by the hospital ward.

- In consultation with the ward there will be a daily check of the medication list on the patient's room. This can be performed during the last medication round. The results of the daily checks should be noted down on the follow up section. Performing a pill count is optional.

- If a patient needs to be re-assessed during a certain time, the result can be noted down on the follow up section. Also, if the patient's health status declines this can be noted down.

Figure S3: The SelfMED procedure.

6 Follow up self-management of medication

The follow up section needs to be completed by the nurses covering the following topics

- **Column 1**: To be completed daily with the date and the nurse's initials

- **Column 2**: The patient self-manages medication: complete on a daily basis
  - If the patient is able to complete their self-medication tick 'YES'
  - If the patient is unable to complete their self-medication state the reason why. Where relevant refer back to reason in section one (nurse assessment) and enter corresponding number. If a new reason, then enter full description.

  After a planned re-evaluation then respond either:
  - Positive re-evaluation based on the criteria directed then enter 'Yes': for example, day 2 after surgery re-evaluation indicated a patient is capable of self-managing medication, thick 'Yes'.
  - Negative re-evaluation based on criteria directed then enter 'No' and the give full details of reason and advise response, where relevant refer back to original evaluation and enter any additional recommendations required.

-**Column 3:** Enter details of visual confirmation of completeness of medication routine as specified in original prescription. If a pill count is performed note down the result. If medication self-management is performed correctly enter 'OK'. If incorrect then note the specific details of the error. For example – incorrect quantity, incorrect timing. Give the specific medication details.

-**Column 4:** If there is any patient deviation from the predetermined criteria and/or prescribed medication, then enter details of any corrective actions that have been implemented, possibly in consultation with the physician. Examples; further education, patient reminders, …

- **Column 5:** If the self-medication can continue then tick 'Continue'. If the self-medication is to be discontinued then tick 'Stop'. Any outstanding medication dosage is then under the control and supervision of the nurse

Figure S3: The SelfMED procedure.

Column 1	Column 2		Column 3		Column 4	Column 5	
**Date + initials nurse**	**Patient capability for self-management**		**Confirmation of completeness of medication administration**		**Intervention**	**SMM**	
	Yes	No, why not?	OK	Not OK + give details of incorrect medication		Conti-nue	Stop

Figure S3: The SelfMED procedure.
