# Supplementary figures and images for: An Evidence-Based Procedure for Self-Management of Medication in Hospital: Development and Validation of the SelfMED Procedure
Source: Pharmacy (Basel). 2018 Jul 26;6(3):77. doi: 10.3390/pharmacy6030077 (PMC6164845; doi:10.3390/pharmacy6030077)

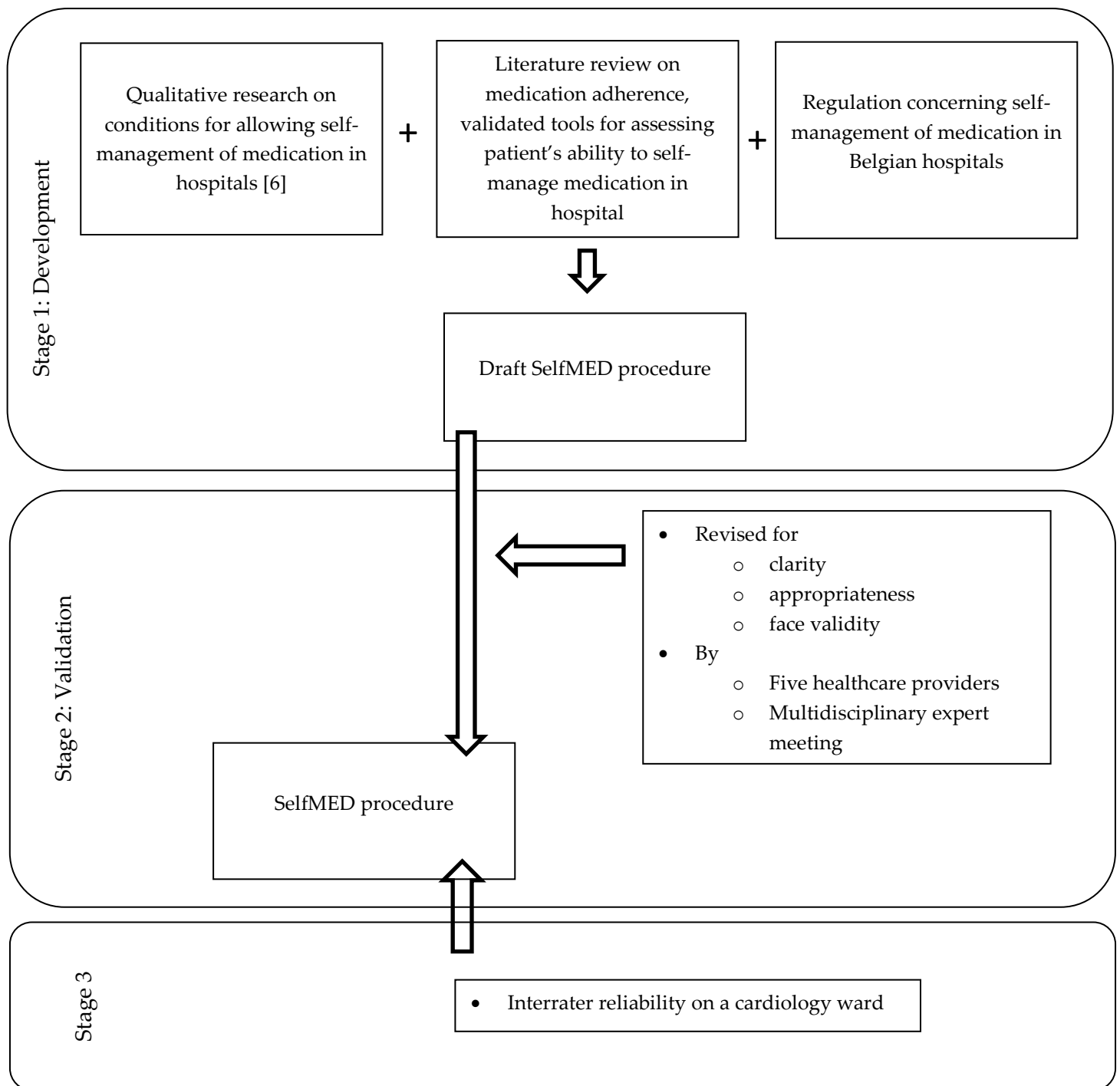

Figure S1: Development and validation of the SelfMED procedure

Supplement: Supplementary file 1 [file pharmacy-06-00077-s001.zip › Supplementary Files/Figure S1.pdf]

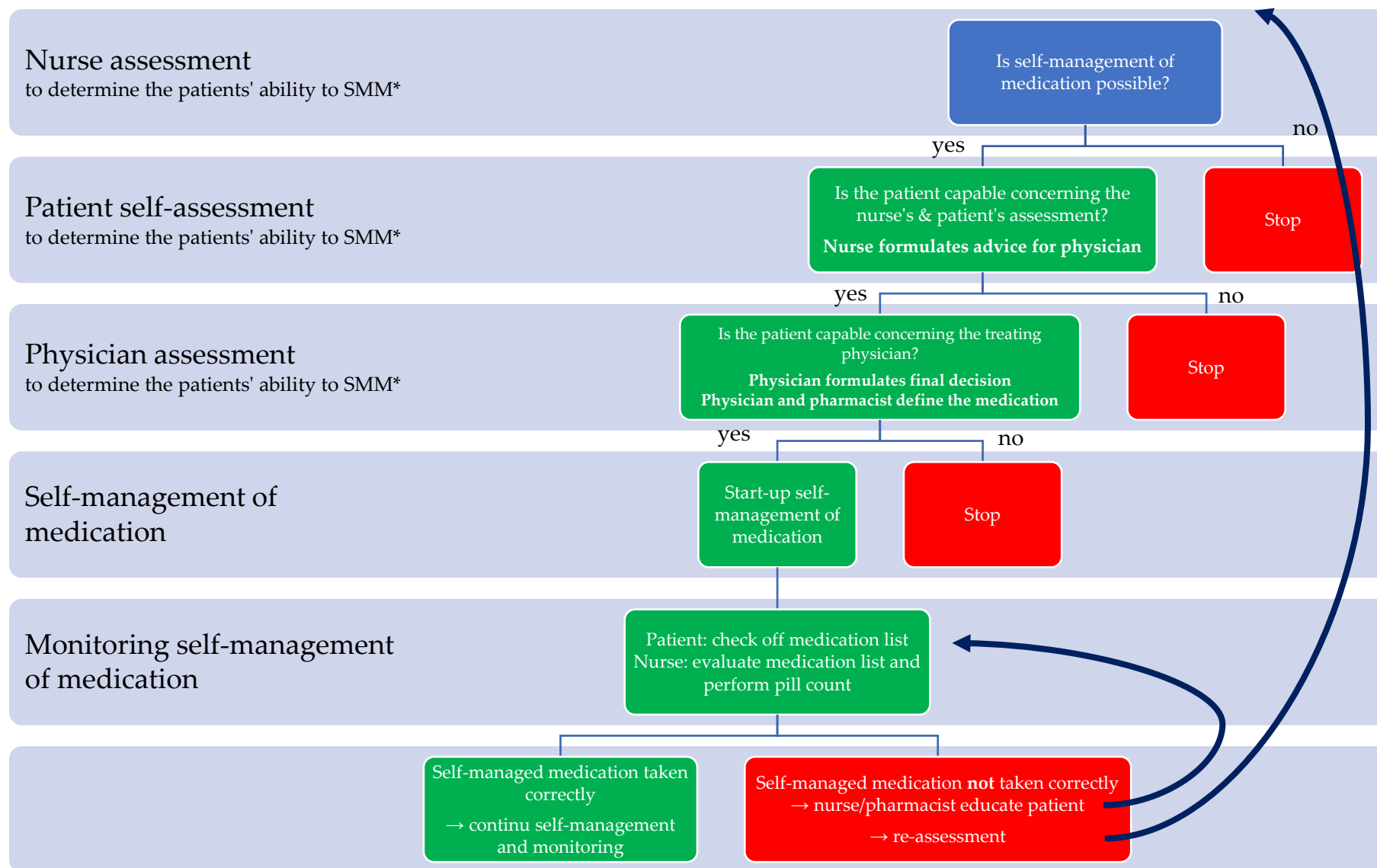

\* SMM: Self-management of medication

Figure S2: The SelfMED flowchart.

Supplement: Supplementary file 1 [file pharmacy-06-00077-s001.zip › Supplementary Files/Figure S2.pdf]
